# Eriophyoid mites (Acari, Eriophyoidea) associated with tea plants, with descriptions of a new genus and two new species

**DOI:** 10.3897/zookeys.534.5961

**Published:** 2015-11-11

**Authors:** Xiao Han, Yun Zuo, Xiao-Feng Xue, Xiao-Yue Hong

**Affiliations:** 1 Department of Entomology, Nanjing Agricultural University, Nanjing, Jiangsu 210095, China

**Keywords:** Prostigmata, *Camellia* spp., Theaceae, taxonomy, Yunnan Province, China

## Abstract

A new genus and two new species of mites in the family Eriophyidae, *Theaphyes rapaneae* **gen. n.** and **sp. n.** which is found on the type host *Rapanea neriifolia* (Sieb. et Zucc.) Mez (Myrsinaceae) and *Paracaphyllisa theacea* **sp. n.**, are described and illustrated. They are vagrants on the tea plant *Camellia sinensis* (L.) Kuntze and no apparent symptoms were detected. A key to the eriophyoid mites including thirteen species associated with tea plants all over the world is provided.

## Introduction

Tea plants (*Camellia*) are perennial evergreen plants in the family Theaceae. They constitute a highly diverse taxon, presently composed of approximately 625 species (The Plant List on-line database 2013), distributed especially in tropical and subtropical areas. Some of these plant species are of extreme socio-economic importance.

Eriophyoid mites (Acari: Prostigmata) have a worldwide distribution. Eriophyoidea is a large mite superfamily with more than 4,000 described species (Zhang et al. 2011). They are strictly phytophagous, many of them can have the pest status in agricultural systems and are considered the second most economically important group of mite plant pests (Lindquist et al. 1996a). Nearly 80% have been reported on a single host species, 95% on one host genus and 99% on one host family (Skoracka et al. 2010).

Between 2009 and 2013, field investigations were conducted in southern part of China in order to look for eriophyoids on tea plants, leading to the discovery of a new genus and two new species. It is worth noting that the new genus and the new species were also found on the type host *Rapanea neriifolia* (Sieb. et Zucc.) Mez (Myrsinaceae).

Along with the current new records, thirteen species have been reported from tea plants worldwide (Table 1). They infest leaves in most cases, occasionally buds, stems and flowers. Some species can cause great economic loss to tea plants. This is the case of *Acaphylla theae* (Watt), a major pest all over the world, inducing leaf russeting, and *Calacarus carinatus* (Green), which causes bronzing and white cast skin streaks on both leaf surfaces. This paper describes the new genus and species, summarizes the main information on the eriophyoid mites found until now on tea plants, and provides a key to these mite species.

**Table 1.** Eriophyoid mites associated with tea trees around the world.

Family	Subfamily	Tribe	Species
Eriophyidae	Cecidophyinae	Colomerini	*Cosetacus camelliae* (Keifer, 1945)
			*Theaphyes rapaneae* **gen. *et* sp. n.**
	Nothopodinae	Colopodacini	*Paracolopodacus camelliae* Kuang & Huang, 1994
	Phyllocoptinae	Acaricalini	*Acaphyllisa indiae* (Keifer, 1954)
			*Acaphyllisa parindiae* Keifer, 1978
			*Acaphylla theae* (Watt, 1898)
			*Acaphylla theavagrans* Kadono, 1992
			*Paracaphyllisa theacea* sp. n.
		Calacarini	*Calacarus carinatus* (Green, 1890)
		Tegonotini	*Phyllocoptacus camelliae* Kuang & Lin, 2005
			*Shevtchenkella camelliae* Song, Xue & Hong, 2008
		Phyllocoptini	*Tergilatus camelliae* Wei, Feng & Huang, 1999
Diptilomiopidae	Diptilomiopinae		*Diptilomiopus camelliae* Wang & Chen, 2013

## Material and methods

Plants were examined in field by the aid of hand-lens (30×) and eriophyoids, together with parts of their host plants, were placed in vials and stored in 75% ethanol. Each vial was marked with the collection data and herbaria were prepared for future identification of plant samples.

In the laboratory, the liquid contents were poured into a Petri dish from the vials, mite specimens were picked up using a fine pin and slide-mounted using Keifer’s Booster and modified Berlese medium (Amrine and Manson 1996). Specimens were examined under a Zeiss A2 (Germany) research microscope equipped with phase contrast (A-plan phase objectives: ×10/0.25, ×20/0.45; EC plan-NEOFLUAR phase objectives: ×40/0.75; ×100/1.3 oil immersion) and drawings were made by camera Lucida. Images were taken with the same microscope (under 100× oil immersion with 10× eyepieces) using an Axio Cam MRc (Carl Zeiss) system, connected to a computer and using Axiovision image analysis software. The morphological terminology follows Lindquist (1996b) and Amrine et al. (2003), and the generic classification was made according to Amrine et al. (2003). The genera elevated after 2003 were arranged in the list by us. Specimens were measured according to de Lillo et al. (2010). For each species, the holotype female measurements precede the corresponding range for paratypes (given in parentheses). All measurements are in micrometers (μm) and are lengths when not otherwise specified. All type specimens are deposited as slide mounted specimens in the Arthropod/Mite Collection of the Department of Entomology, Nanjing Agricultural University (NJAU), Jiangsu Province, China.

Host plant names and their synonymies are in accordance with The Plant List (http://www.theplantlist.org/). Data on eriophyoid mites were extracted from the catalogue by Davis et al. (1982), Amrine and Stasny (1994) and from the computerized catalog of the Eriophyoidea (Amrine and de Lillo, pers. comm.); a further record was added based on the searching made on the most common abstract indexes. When available, the following information was summarized for each listed eriophyoid species based on literature: a) previous genus name assignment and possible synonymies; b) information about the host plant species; c) documenting relationships between eriophyoid species and host plants based on literature; d) distribution within geographic realms according to Udvardy (1975); e) the most relevant remarks.

## Results

Thirteen eriophyoid mite species in eleven genera of two eriophyoid families have been reported from tea plants around the world (Table 1) and a key to eriophyoid mites on tea plants is provided below.

### Theaphyes gen. n.

Taxon classificationAnimaliaProstigmataEriophyidae

http://zoobank.org/7929EC1E-07C5-47E5-AA24-D67734AC5E6D

#### Type species.

*Theaphyes rapaneae* sp. n.

#### Diagnosis.

Body fusiform; scapular tubercles placed ahead of rear shield margin and scapular setae projecting upwards; frontal shield lobe absent. All coxal setae present; antaxial genual seta absent from leg II; tarsal solenidion slightly knobbed, located below empodium; empodium entire. Opisthosoma with a wide dorsal furrow; all usual opisthosoma setae present with the exception of setae *e* and *h1*; female genital cover flap appressed to coxal plates.

#### Etymology.

The genus designation is the combination of *Thea*- and -*phyes*; *Thea*- is derived from the family name of the host plant, -*phyes* is derived from the type genus *Eriophyes* in the family Eriophyidae. The gender is feminine.

#### Remarks.

The new genus is assigned to the Family Eriophyidae, Subfamily Cecidophyinae, Tribe Colomerini. It is similar to *Epicecidophyes clerodendris* Mondal & Chakrabarti, 1981, but can be differentiated from the latter by the absence of opisthosomal setae *e* (setae *e* are present in *Epicecidophyes*) and by a wide furrow on the dorsal opisthosoma (a broad middorsal ridge is on the dorsal opisthosoma of *Epicecidophyes*). The tarsal solenidion is located below the empodium similarly to *Catachela machaerii* Keifer, 1969, *Dechela epelis* Keifer, 1965 and *Dechela phoebe* Wang, Han, Xue & Hong, 2014.

### Theaphyes rapaneae sp. n.

Taxon classificationAnimaliaProstigmataEriophyidae

http://zoobank.org/B7A8BE17-72C9-4524-BFB9-2E2C7CFBCE86

Fig. 1

#### Description.

FEMALE: (n = 9, ventral-dorsal position on slides). Body fusiform, white, 152 (152–153), 80 (80–85) wide. **Gnathosoma** 19 (19–20), projecting obliquely downwards, pedipalp coxal setae (*ep*) 2 (2–3), dorsal pedipalp genual setae (*d*) 8 (7–8), cheliceral stylets 20 (19–20). **Prodorsal shield** 58 (57–58), 80 (80–82) wide, admedian lines complete curving mesally at their posterior ends; frontal shield lobe absent. Scapular tubercles ahead of rear shield margin, 25 (24–25) apart, scapular setae (*sc*) 2 (2–3), projecting upward. **Coxigenital region** with 5 (5–6) semiannuli between coxae and genitalia. Coxal plates with a few short lines, anterolateral setae on coxisternum I (*1b*) 7 (6–7), 15 (15–16) apart, proximal setae on coxisternum I (*1a*) 11 (10–11), 14 (14–15) apart, proximal setae on coxisternum II (*2a*) 12 (12–15), 33 (32–33) apart. Prosternal apodeme absent. **Leg I** 27 (26–27), femur 11 (10–11), basiventral femoral setae (*bv*) 5 (5–6); genu 3 (2–3), antaxial genual setae (*l*’’) 21 (20–21); tibia 3 (2–3), paraxial tibial setae (*l*’) 4 (4–5), located at 2/3 from the dorsal base; tarsus 7 (6–7), paraxial, fastigial, tarsal setae (*ft*’) 15 (15–16), antaxial, fastigial, tarsal setae (*ft*’’) 18 (18–20), paraxial, unguinal, tarsal setae (*u*’) 4 (4–5); tarsal empodium (*em*) 5 (4–5), simple, 5-rayed, tarsal solenidion (*ω*) 5 (5–6), slightly knobbed, located below empodium. **Leg II** 20 (20–21), femur 10 (10–11), basiventral femoral setae (*bv*) 6 (6–7); genu 3 (2–3), antaxial genual setae (*l*’’) absent; tibia 2 (2–3); tarsus 7 (6–7), paraxial, fastigial, tarsal setae (*ft*’) 3 (3–4), antaxial, fastigial, tarsal setae (*ft*’’) 18 (17–18), paraxial, unguinal, tarsal setae (*u*’) 4 (3–4); tarsal empodium (*em*) 5 (4–5), simple, 5-rayed, tarsal solenidion (*ω*) 7 (7–8), little knobbed, located below empodium. **Opisthosoma** dorsally with 26 (26–29) semiannuli, smooth, with a wide furrow, ventrally with 54 (52–54) semiannuli, with linear microtubercles. Setae *c2* 19 (18–19) on ventral semiannulus 6 (6–7), 85 (80–85) apart; setae *d* 35 (34–35) on ventral semiannulus 21 (20–21), 32 (30–32) apart; setae *e* absent, setae *f* 15 (15–16) on 5th–6th ventral semiannulus from rear, 20 (20–21) apart. Setae *h1* absent, *h2* 50 (50–60). **Genital coverflap** 20 (18–20), 38 (38–39) wide, coverflap with 22–23 longitudinal ridges in one rank, some ridges not complete, setae *3a* 6 (5–6), 24 (18–24) apart.

MALE: (n = 4, ventral-dorsal position on slides). 142 (142–145), 69 (69–70) wide; white. **Gnathosoma** 19 (18–19), projecting obliquely downwards, pedipalp coxal setae (*ep*) 2 (1–2), dorsal pedipalp genual setae (*d*) 6 (5–6), cheliceral stylets 17 (16–17). **Prodorsal shield** 53 (53–54), 65 (65–70) wide, admedian lines complete curving mesally at their posterior ends; frontal shield lobe absent. Scapular tubercles ahead of rear shield margin, 22 (22–24) apart, scapular setae (*sc*) 3 (2–3), projecting upward. **Coxigenital region** with 5 (5–6) semiannuli between coxae and genitalia. Coxal plates with a few short lines, anterolateral setae on coxisternum I (*1b*) 5 (5–6), 10 (10–12) apart, proximal setae on coxisternum I (*1a*) 7 (7–8), 14 (12–14) apart, proximal setae on coxisternum II (*2a*) 10 (10–11), 27 (27–28) apart. Prosternal apodeme absent. **Leg I** 20 (20–21), femur 12 (11–12), basiventral femoral setae (*bv*) 5 (5–6); genu 3 (2–3), antaxial genual setae (*l*’’) 17 (17–18); tibia 3 (2–3), paraxial tibial setae (*l*’) 4 (4–5), located at 2/3 from the dorsal base; tarsus 5 (4–5), paraxial, fastigial, tarsal setae (*ft*’) 13 (13–14), antaxial, fastigial, tarsal setae (*ft*’’) 17 (15–17), paraxial, unguinal, tarsal setae (*u*’) 4 (4–5); tarsal empodium (*em*) 5 (4–5), simple, 5-rayed, tarsal solenidion (*ω*) 5 (5–6), slightly knobbed, located below empodium. **Leg II** 18 (17–18), femur 9 (8–9), basiventral femoral setae (*bv*) 4 (4–6); genu 2 (2–3), antaxial genual setae (*l*’’) absent; tibia 3 (3–4); tarsus 5 (4–5), paraxial, fastigial, tarsal setae (*ft*’) 4 (4–5), antaxial, fastigial, tarsal setae (*ft*’’) 15 (14–15), paraxial, unguinal, tarsal setae (*u*’) 3 (3–4); tarsal empodium (*em*) 5 (4–5), simple, 5-rayed, tarsal solenidion (*ω*) 7 (7–8), slightly knobbed, located below empodium. **Opisthosoma** dorsally with 28 (26–28) semiannuli, smooth, with a wide furrow; ventrally with 51 (48–51) semiannuli, with linear microtubercles. Setae *c2* 12 (12–13) on ventral semiannulus 6 (5–6), 65 (64–69) apart; setae *d* 20 (18–22) on ventral semiannulus 16 (15–17), 27 (25–27) apart; setae *e* absent, setae *f* 14 (14–15) on 5th–6th ventral semiannulus from rear, 20 (18–20) apart. Setae *h1* absent, *h2* 52 (51–52). Genitalia 20 (20–21) wide, setae *3a* 5 (5–6), 15 (13–15) apart.

**Figure 1.**
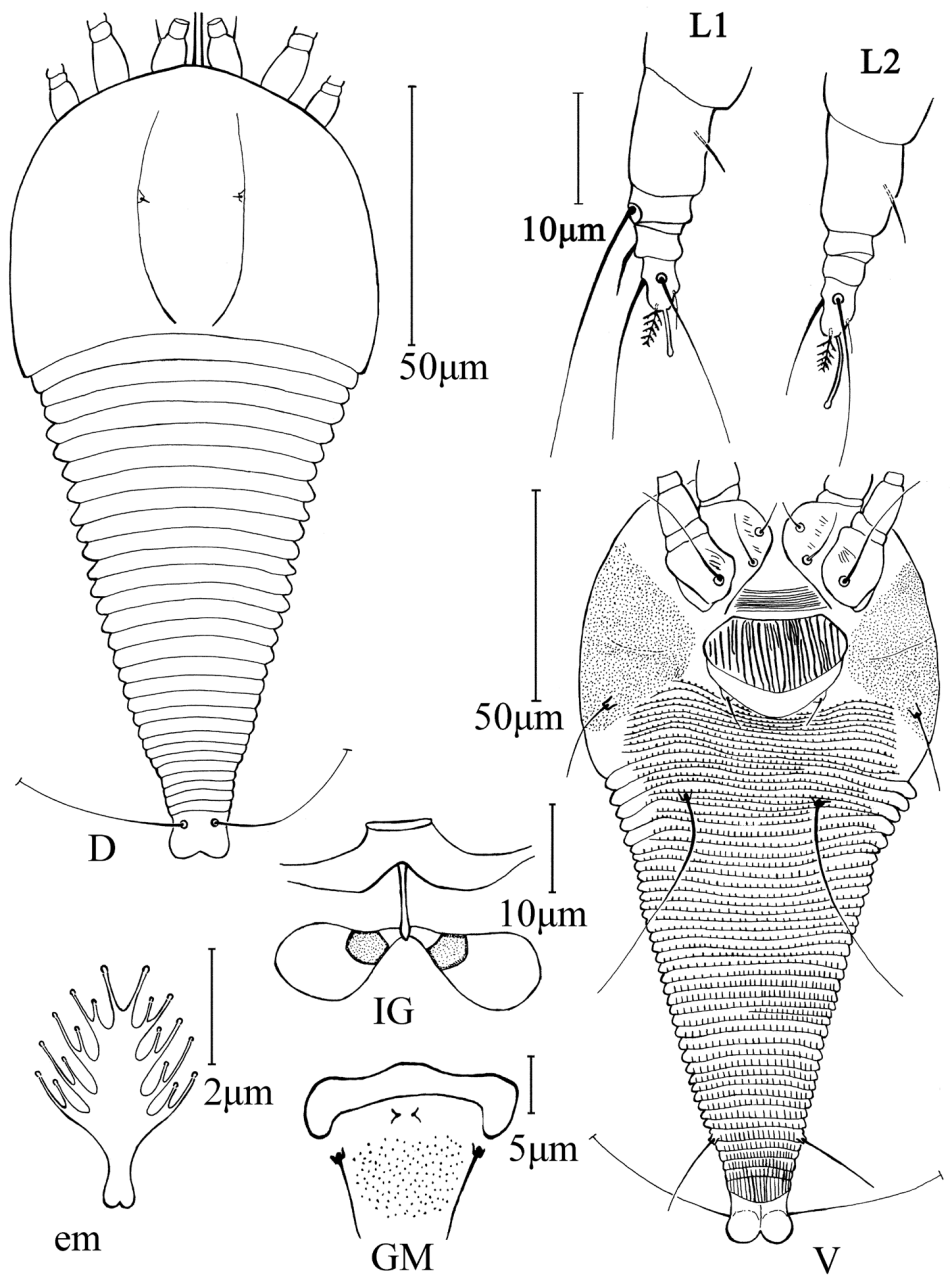
*Theaphyes rapaneae* sp. n.: **D** dorsal view of female **V** ventral view of female **em** empodium **IG** female internal genitalia **GM** male genital region **L1** leg I **L2** leg II.

#### Type host plant.

*Rapanea neriifolia* (Sieb. et Zucc.) Mez (Myrsinaceae)

#### Other host plant.

*Camellia sinensis* (L.) Kuntze

#### Relation to the host plant.

Vagrant. No damage to the host plant was observed.

#### Type locality.

Nanling National Forest Park, Guangdong Province (24°53'50"N, 113°01'18"E), elevation 1,408 m, 31 July 2012, coll. Qiong Wang, Hao-Sen Li and Jing-Feng Guo.

#### Type material.

Holotype, single female on a microscope slide (slide number NJAUAcariEriGD21.1; marked Holotype), from *Rapanea neriifolia*. Paratypes, 4 females and 4 males mounted on separate microscope slides (slide number NJAUAcariEriGD21.2–NJAUAcariEriGD21.9), same collection data of the holotype.

#### Other material.

10 females mounted on separate microscope slides (slide numbers NJAUAcariEriYN304B.1–NJAUAcariEriYN304B.10), from *Camellia sinensis*.

#### Etymology.

The specific designation *rapaneae* is the genitive case derived from the genus name of the type host plant, *Rapanea*.

#### Remarks.

This species is found on the Myrsinaceae *Rapanea neriifolia* and also on *Camellia sinensis*. Slight morphological differences were observed between the populations found on the two host species: the population on *Camellia sinensis* is longer, thinner and with more ventral annuli (62–66) than the population on *Rapanea neriifolia* which is provided with fewer ventral annuli (52–54). The population found on *Camellia sinensis* occurred with other eriophyoid species and no males were collected. Current data do not allow understanding if the mite species colonizes regularly and successfully *Camellia sinensis* and it needs further biological studies.

### Paracaphyllisa theacea sp. n.

Taxon classificationAnimaliaProstigmataEriophyidae

http://zoobank.org/AC45840F-0982-40F3-8897-CDD76B3FD531

Fig. 2

#### Description.

FEMALE: (n = 8, ventral-dorsal position on slides). Body fusiform, 200 (190–200), 80 (75–80) wide; white. **Gnathosoma** 35 (32–35), projecting obliquely downwards, pedipalp coxal setae (*ep*) 3 (2–3), dorsal pedipalp genual setae (*d*) 9 (8–9), cheliceral stylets 40 (38–40). **Prodorsal shield** 50 (50–52), 75 (70–75) wide, median line absent, admedian and submedian lines sinuous; front shield lobe present 12 (11–12). Scapular tubercles ahead of rear shield margin, 37 (35–37) apart, scapular setae (*sc*) 4 (3–4), projecting anteriorly. **Coxigenital region** with 8 (8–9) semiannuli between coxae and genitalia, smooth. Coxal plates smooth, anterolateral setae on coxisternum I (*1b*) 10 (10–11), 14–15 apart, anterolateral setae on coxisternum I (*1a*) 15 (13–15), 12 (11–12) apart, proximal setae on coxisternum II (*2a*) 40 (35–40), 30 (30–31) apart. Prosternal apodeme absent. **Leg I** 37 (37–40), femur 15 (14–15), basiventral femoral setae (*bv*) 15 (15–16); genu 5 (4–5), antaxial genual setae (*l*’’) 43 (42–43); tibia 8 (7–8), paraxial tibial setae (*l*’) 1, located at center; tarsus 8 (7–8), paraxial, fastigial, tarsal setae (*ft*’) 30 (29–30), antaxial, fastigial, tarsal setae (*ft*’’) 33 (33–34), paraxial, unguinal, tarsal setae (*u*’) 6 (5–6); tarsal empodium (*em*) 5 (5–6), divided, 5-rayed, tarsal solenidion (*ω*) 12 (11–12), slightly knobbed. **Leg II** 34 (32–34), femur 12 (12–14), basiventral femoral setae (*bv*) 11 (10–11); genu 5 (4–5), antaxial genual setae (*l*’’) absent; tibia 6 (5–6); tarsus 8 (7–8), paraxial, fastigial, tarsal setae (*ft*’) 9 (9–10), antaxial, fastigial, tarsal setae (*ft*’’) 26 (26–27), paraxial, unguinal, tarsal setae (*u*’) 5 (4–5); tarsal empodium (*em*) 5 (5–6), divided, 5-rayed, tarsal solenidion (*ω*) 11 (11–12), slightly knobbed. **Opisthosoma** dorsally with 38 (37–38) semiannuli, smooth, with three ridges, ventrally with 62 (62–69) semiannuli, with rounded microtubercles. Setae *c2* 35 (33–35) on ventral semiannulus 15 (12–15), 60 (57–60) apart; setae *d* 70 (67–70) on ventral semiannulus 26 (25–27), 45 (43–45) apart; setae *e* 50 (50–52) on ventral semiannulus 41 (41–44), 20 (19–20) apart, setae *f* 23 (23–25) on 6th–7th ventral semiannulus from rear, 15 (15–16) apart. Setae *h1* absent, *h2* 55 (52–55). **Genital coverflap** 15 (15–16), 30 (29–30) wide, coverflap with 23 (18–23) longitudinal ridges and dense short lines at base, setae *3a* 14 (13–14), 20 (20–21) apart.

MALE: Unknown.

**Figure 2.**
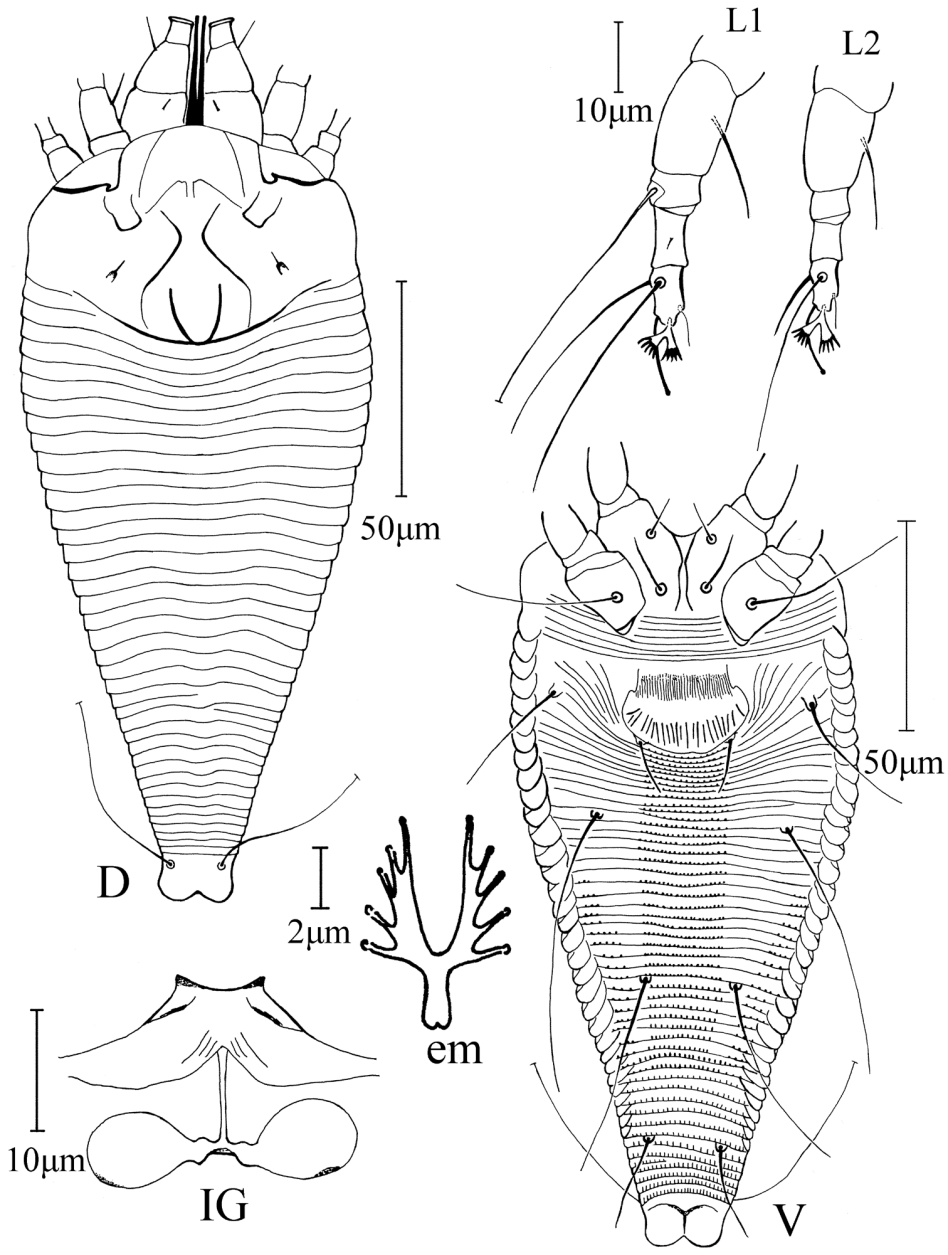
*Paracaphyllisa theacea* sp. n.: **D** dorsal view of female **V** ventral view of female **em** empodium **IG** female internal genitalia **L1** leg I **L2** leg II.

#### Type host plant.

*Camellia sinensis* (L.) Kuntze

#### Relation to the host plant.

Vagrant. No damage to the host plant was observed.

#### Type locality.

Pihe Village, Fugong County, Nujiang Lisu autonomous prefecture, Yunnan Province (26°33'05"N, 98°55'08"E), elevation 2,122 m, 26 June 2013, coll. Xiao Han, Qiong Wang and Jing-Feng Guo.

#### Type material.

Holotype, single female on a microscope slide (slide number NJAUAcariEriYN304C.1; marked Holotype). **Paratypes** 7 females mounted on separate microscope slides (slide number NJAUAcariEriYN304C.2–NJAUAcariEriYN304C.8).

#### Etymology.

The specific designation *theacea* is derived from the family name of the host plant; feminine in gender.

#### Differential diagnosis.

This new species is similar to *Paracaphyllisa adinandrae* Kuang & Luo, 2005, but can be differentiated from the latter by the design of prodorsal shield which is provided with admedian and submedian lines (prodorsal shield design of *Paracaphyllisa adinandrae* has median, admedian and submedian lines), smooth coxal plates (coxal plates have short lines in *Paracaphyllisa adinandrae*) and coverflap with 23 (18–23) longitudinal ridges and dense short lines at its base (coverflap is smooth in *Paracaphyllisa adinandrae*).

#### Remarks.

The new species is surrounded with white hairs around the body.

### Accounts of further species on *Camellia*

#### Acaphyllisa indiae

Taxon classificationAnimaliaProstigmataEriophyidae

(Keifer, 1954)

Acaphylla indiae ; Keifer 1954: 126.
Acaphyllisa indiae ; Amrine and Stasny 1994: 14.

##### Host.

*Camellia sinensis* (L.) Kuntze.

##### Relation to the host plant.

Vagrant, causing leaf rusting.

##### Distribution.

Indomalayan region.

#### Acaphyllisa parindiae

Taxon classificationAnimaliaProstigmataEriophyidae

Keifer, 1978

Acaphyllisa parindiae ; Keifer 1978: 15.

##### Host.

*Camellia sinensis* (L.) Kuntze.

##### Relation to the host plant.

Vagrant, causing leaf rusting.

##### Distribution.

Indomalayan region.

#### Acaphylla theae

Taxon classificationAnimaliaProstigmataEriophyidae

(Watt, 1898)

Phytoptus theae Watt, 1898
Eriophyes theae (Watt, 1903); Nalepa 1923: 46.
Acaphylla theae ; Das and Sengupta 1958: 40 (synonym of *Acaphylla steinwedeni* Keifer, 1943).
Acaphylla steinwedeni ; Keifer 1943: 215.
Acaphylla steinwedeni ; Keifer 1954: 126.
Acaphylla steinwedeni ; Keifer 1975: 545.
Acaphylla steinwedeni ; Amrine and Stasny 1994: 13.
Acaphylla steinwedeni ; Baker et al. 1996: 86.

##### Host.

*Camellia japonica* L., *Camellia oleifera* Abel, *Camellia reticulata* Lindl., *Camellia sasanqua* Thunb., *Camellia sinensis* (L.) Kuntze, *Camellia sinensis* var. *assamica* (J.W. Mast.) Kitam.

##### Relation to the host plant.

Vagrant. This species occurs on the undersurface of the leaves and is often associated with *Calacarus carinatus* (Green) (reported as *Camellia adornatus* (Keifer, 1940)). *Acaphylla steinwedeni* does not leave as much debris on the leaves as *Calacarus carinatus*, but it may actually be more important as a rust mite. Both mite species overwinter on the leaves and show no deuterogyny.

##### Distribution.

Australian, Nearctic, Indomalayan and Palaearctic regions.

##### Remarks.

*Phytoptus theae* was reported infesting *Camellia sinensis* by Watt (1898) who provided a drawing and a short description of the mite, and described the injury on the tea plant. Many authors have wrongfully listed the mite as *Phytoptus theae* Watt & Mann, 1903, based on the more available publication. Improperly, Nalepa (1929) listed *Phytoptus theae* as a nude name. Keifer (1954) identified *Acaphylla steinwedeni* from specimens provided by Dr. Das and which were referred to have been the object of the name *Phytoptus theae*. Das and Sengupta (1958) made *Acaphylla steinwedeni* as junior synonym of *Acaphylla theae*, making this last one as the genotype of the genus *Acaphylla*. Finally, Amrine and Stasny (1994) listed *Acaphylla theae* as possible synonym (they marked the synonymy with a question mark) of *Acaphylla steinwedeni* without any further indication about it and the use of the names.

#### Acaphylla theavagrans

Taxon classificationAnimaliaProstigmataEriophyidae

Kadono, 1992

Acaphylla theavagrans ; Kadono 1992: 149–151.

##### Host.

*Camellia sinensis* (L.) Kuntze.

##### Relation to the host plant.

Vagrant, causing rust.

##### Distribution.

Indomalayan region.

#### Calacarus carinatus

Taxon classificationAnimaliaProstigmataEriophyidae

(Green, 1890)

Typhlodromus carinatus Green, 1890: 35.
Eriophyes carinatus ; Nalepa 1923: 31.
Epitrimerus adornatus ; Keifer 1940: 32.
Calacarus carinatus ; Baker et al. 1996: 86.

##### Host.

*Camellia caudata* Wallich, *Camellia sinensis* (L.) Kuntze, *Camellia kissi* Wallich, *Camellia japonica* L., *Camellia sasanqua* Thunb., *Capsicum annuum* L. (Solanaceae), *Viburnum opulus* L. (Adoxaceae).

##### Relation to the host plant.

Vagrant, causing bronzing and leaving white cast skin streaks. Wax is produced on the five ridges and prodorsal shield.

##### Distribution.

Africotropical, Australian, Indomalayan, Nearctic, Palaearctic regions.

##### Remarks.

This free-living species leaves much debris on the host leaves and occurs on the leaves associated with *Acaphylla steinwedeni*. They both overwinter on the leaves and show no deuterogyny.

#### Cosetacus camelliae

Taxon classificationAnimaliaProstigmataEriophyidae

(Keifer, 1945)

Aceria camelliae ; Keifer 1945: 137–138.
Cosetacus camelliae ; Amrine and Stasny 1994: 168.
Cosetacus camelliae ; Baker et al. 1996: 86.

##### Host.

*Camellia japonica* L., *Camellia* sp.

##### Relation to the host plant.

Vagrant. The mite lives under leaf and flower buds, probably causing premature flower drop.

##### Distribution.

Australian, Nearctic, Neotropic, Palaearctic regions.

#### Diptilomiopus camelliae

Taxon classificationAnimaliaProstigmataDiptilomiopidae

Wang & Chen, 2013

Diptilomiopus camelliae ; Tan et al. 2013: 802–804.

##### Host.

*Camellia caudata* Wallich.

##### Relation to the host plant.

Vagrant. No damage to the host was observed.

##### Distribution.

On the border between Palaearctic and Indomalayan regions.

#### Paracolopodacus camelliae

Taxon classificationAnimaliaProstigmataEriophyidae

Kuang & Huang, 1994

Paracolopodacus camelliae ; Kuang and Huang 1994: 229–230.

##### Host.

*Camellia oleifera* Abel.

##### Relation to the host plant.

Vagrant.

##### Distribution.

On the border between Palaearctic and Indomalayan regions.

#### Phyllocoptacus camelliae

Taxon classificationAnimaliaProstigmataEriophyidae

Kuang & Lin, 2002

Phyllocoptacus camelliae ; Kuang and Lin 2002: 84–85.
Phyllocoptacus camelliae ; Kuang et al. 2005: 51–52.

##### Host.

*Camellia sinensis* (L.) Kuntze.

##### Relation to the host plant.

Vagrant.

##### Distribution.

On the border between Palaearctic and Indomalayan regions.

#### Shevtchenkella camelliae

Taxon classificationAnimaliaProstigmataEriophyidae

Song, Xue & Hong, 2008

Shevtchenkella camelliae ; Song et al. 2008: 48–49.

##### Host.

*Camellia sinensis* (L.) Kuntze.

##### Relation to the host plant.

Vagrant, causing no apparent damage to the host plant.

##### Distribution.

On the border between Palaearctic and Indomalayan regions.

#### Tergilatus camelliae

Taxon classificationAnimaliaProstigmataEriophyidae

Wei, Feng & Huang, 1999

Tergilatus camelliae ; Wei et al. 1999: 144–146.

##### Host.

*Camellia sinensis* (L.) Kuntze.

##### Relation to the host plant.

Not stated.

##### Distribution.

On the border between Palaearctic and Indomalayan regions.

### Key to eriophyoid mite species associated with tea plant

**Table:** 

1	Gnathosoma small in comparison to the body, chelicerae straight or slightly curved	**2**
–	Gnathosoma large in comparison to the body, chelicerae abruptly curved and bent down near their base	***Diptilomiopus camelliae* Wang & Chen, 2013**
2	Tibiae reduced or completely fused with tarsi	***Paracolopodacus camelliae* Kuang & Huang, 1994**
–	Tibiae distinct from tarsi	**3**
3	Setae *e* absent	**4**
–	Setae *e* present	**5**
4	Median line absent, admedian lines complete curving mesally at their posterior ends; frontal lobe absent. Scapular setae *sc* projecting upward. Empodium 5-rayed	***Theaphyes rapaneae* gen. et sp. n.**
–	Median, admedian and submedian lines incomplete; frontal lobe present. Scapular setae *sc* projecting upward. Empodium 6-rayed	***Tergilatus camelliae* Wei, Feng & Huang, 1999**
5	Female genital apodeme bent up and shortened, usually appearing as a heavy transverse line in ventral view, ridges on female coverflap in 2 uneven ranks	***Cosetacus camelliae* (Keifer, 1945)**
–	Female genital apodeme extending moderate distance forward, does not appear as a heavy transverse bar in ventral view, female coverflap smooth or variably sculptured	**6**
6	Empodium entire	**7**
–	Empodium divided	**9**
7	Scapular tubercles and setae *sc* absent	***Calacarus carinatus* Green, 1890**
–	Scapular tubercles and setae *sc* present	**8**
8	Dorsal opisthosoma with anterior annuli fused forming a broad plate joined to prodorsal shield. Prodorsal shield with admedian and submedian lines, scapular tubercles ahead of rear shield margin, scapular setae *sc* projecting upward. Leg II without genual setae (*l*’’), tarsal empodium 6-rayed. Coverflap smooth	***Phyllocoptacus camelliae* Kuang & Lin, 2005**
–	Dorsal opisthosoma without fused annuli forming a plate. Prodorsal shield smooth, scapular tubercles on rear shield margin, scapular setae *sc* projecting posteriorly. Leg II with usual setae, tarsal empodium 7-rayed. Coverflap with 14 longitudinal ridges	***Shevtchenkella camelliae* Song, Xue & Hong, 2008**
9	Coxal setae *1b* absent	**10**
–	Coxal setae *1b* present	**11**
10	Coxal area with short lines, prodorsal shield with median line present on the posterior 2\5 and 4\5, frontal lobe bilobed, coxae smooth	***Acaphylla theae* (Watt, 1898)**
–	Coxal area smooth, prodorsal shield with median line absent, frontal lobe not as above, coxae with granules	***Acaphylla theavagrans* Kadono, 1992**
11	Genual II setae (*l*”) absent	**12**
–	Genual II setae (*l*”) present; prodorsal shield without median line, admedian lines with short recurving sections, meeting cross lines at 1/4 and 2/3, tarsal empodium 3-rayed	***Acaphyllisa parindiae* Keifer, 1978**
12	Prodorsal shield with median line complete, submedian lines curving from the median and forming a double loop between the dorsal tubercles, prodorsal shield laterally with a broad lobe over the coxae. Leg II with femoral seta *(bv)* absent, tarsal empodium 8-rayed	***Acaphyllisa indiae* Keifer, 1954**
–	Prodorsal shield without median line, submedian lines not as above, prodorsal shield laterally without a broad lobe over the coxae. Leg II with femoral seta *(bv)* present, tarsal empodium 5-rayed	***Paracaphyllisa theacea* sp. n.**

## Supplementary Material

XML Treatment for Theaphyes

XML Treatment for Theaphyes rapaneae

XML Treatment for Paracaphyllisa theacea

XML Treatment for Acaphyllisa indiae

XML Treatment for Acaphyllisa parindiae

XML Treatment for Acaphylla theae

XML Treatment for Acaphylla theavagrans

XML Treatment for Calacarus carinatus

XML Treatment for Cosetacus camelliae

XML Treatment for Diptilomiopus camelliae

XML Treatment for Paracolopodacus camelliae

XML Treatment for Phyllocoptacus camelliae

XML Treatment for Shevtchenkella camelliae

XML Treatment for Tergilatus camelliae

## References

[B1] Amrine JW Jr Manson DCM (1996) Preparation, mounting and descriptive study of eriophyoid mites. In: Lindquist EE Sabelis MW Bruin J (Eds) Eriophyoid Mites: Their Biology, Natural Enemies and Control. Elsevier, World Crop Pests 6, Elsevier Science Publishers, Amsterdam Netherlands, 383–396. doi: 10.1016/S1572-4379(96)80023-6

[B2] Amrine JW Jr Stasny TA (1994) Catalog of the Eriophyoidea (Acarina: Prostigmata) of the world. Indira Publishing House, West Bloomfield, Michigan, USA, 804 pp.

[B3] Amrine JW Jr Stasny TA Flechtmann CHW (2003) Revised keys to world genera of Eriophyoidea (Acari: Prostigmata). Indira Publishing House, West Bloomfield, Michigan, USA, 244 pp.

[B4] Baker EW Kono T Amrine JW Jr Delfinado-Baker M Stasny TA (1996) Eriophyoid mites of the United States. Indira Publishing House, West Bloomfield, Michigan, USA, 394 pp.

[B5] Das GM Sengupta N (1958) Observations on the pink mite, *Acaphylla theae* (Watt) Keifer, of tea in north-east India. Journal of Zoological Society, India 10: 39–48.

[B6] Davis R Flechtmann CHW Boczek JH Barke HE (1982) Catalogue of Eriophyid Mites (Acari, Eriophyoidea). Warsaw Agricultural University Press, Warsaw, Poland, 254 pp.

[B7] de Lillo E Craemer C Amrine JW Jr Nuzzaci EG (2010) Recommended procedures and techniques for morphological studies of Eriophyoidea (Acari: Prostigmata). Experimental and Applied Acarology 51(1–3): 283–307. doi: 10.1007/s10493-009-9311-x 19771397 10.1007/s10493-009-9311-x

[B8] Green EE (1890) Insect Pests of the tea plant. Colombo, Ceylon, 85 pp.

[B9] Kadono F (1992) A new species of eriophyid mite injurious to tea plant in Japan (Acari: Eriophyidae). Acta Arachnologica 41(2): 149–152. doi: 10.2476/asjaa.41.149

[B10] Keifer HH (1940) Eriophyid Studies VIII. Bulletin of the California Department of Agriculture 29: 21–46.

[B11] Keifer HH (1943) Eriophyid Studies XIII. Bulletin of the California Department of Agriculture 32: 212–222.

[B12] Keifer HH (1945) Eriophyid Studies XV. Bulletin of the California Department of Agriculture 34: 137–140.

[B13] Keifer HH (1954) Eriophyid Studies XXII. Bulletin of the California Department of Agriculture 43: 121–131.

[B14] Keifer HH (1965) Eriophyid Studies B-13. Bulletin of the California Department of Agriculture, 1–20.

[B15] Keifer HH (1969) Eriophyid Studies C-2. Agriculture Research Service-United State Department of Agriculture, 1–20.

[B16] Keifer HH (1975) Injurious eriophyoid mites. In: Jeppson LR Keifer HH Baker EW Mites injurious to economic plants. University California Press, Berkeley, California, USA, 327–561.

[B17] Keifer HH (1978) Eriophyid Studies C-15. Agriculture Research Service-United State Department of Agriculture, 1–24.

[B18] Kuang HY Huang LW (1994) A new genus and two new species of Eriophyidae from China (Acari: Eriophyoidea). Acta Entomologica Sinica 37(2): 229–232.

[B19] Kuang HY Luo GH Wang AW (2005) Fauna of eriophyid mites from China (II) (Acari: Eriophyoidea). China Forestry Publishing House, Beijing, China, 176 pp.

[B20] Kuang HY Lin JZ (2002) A new species of pest mite on tea in China *Phyllocoptacus camelliae* Kuang & Lin sp. n. (Acari: Eriophyoidea). Journal of Tea 28(2): 84–85. [In Chinese, with English abstract]

[B21] Lindquist EE (1996a) Evolution of Eriophyoid mites in relation to their host plants. In: Lindquist EE Sabelis MW Bruin J (Eds) Eriophyoid mites: their biology, natural enemies and control. Elsevier, World Crop Pests, 6, Elsevier Science Publishers, Amsterdam, Netherlands, 277–300. doi: 10.1016/S1572-4379(96)80018-2

[B22] Lindquist EE (1996b) External anatomy and notation of structures. In: Lindquist EE Sabelis MW Bruin J (Eds) Eriophyoid mites: their biology, natural enemies and control. World Crop Pests, 6, Elsevier Science Publishers, Amsterdam, Netherlands, 3–31. doi: 10.1016/S1572-4379(96)80003-0

[B23] Mondal S Chakrabarti S (1981) Studies on eriophyid mites (Acarina: Eriophyoidea) of India. X. New genus and new species from West Bengal. Oriental Insects 15(3): 313–319. doi: 10.1080/00305316.1981.10434400

[B24] Nalepa A (1923) Index nominum quae ab a. 1886 eriophyidarum generibus, speciebus et subspecibus imposita sunt. Conscriptus ab. Marcellia 20: 25–66.

[B25] Nalepa A (1929) Neuer Katalog der bisher Beschriebenen Gallmilben, ihrer Gallen und Wirtspflanzen. Marcellia 25: 67–183.

[B26] Skoracka A Smith L Oldfield G Cristofaro M Amrine JW Jr (2010) Host-plant specificity and specialization in eriophyoid mites and their importance for the use of eriophyoid mites as biocontrol agents of weeds. Experimental and Applied Acarology 51(1–3): 93–113. doi: 10.1007/s10493-009-9323-6 19789985 10.1007/s10493-009-9323-6

[B27] Song ZW Xue XF Hong XY (2008) One new genus and four new species of Phyllocoptinae (Acari: Eriophyoidea) from Fujian Province, southeastern China. Zootaxa 1894: 42–52.

[B28] Tan Z Lu XQ Tao LF Chen ZJ Wang GQ (2013) Four new species of the genus *Diptilomiopus* Nalepa from China (Acari, Eriophyoidea). Acta Zootaxonomica Sinica 38(4): 801–806.

[B29] The Plant List (2013) Version 1.1. Published on the Internet. http://www.theplantlist.org/

[B30] Udvardy MDF (1975) A Classification of the Biogeographical Provinces of the World. International Union for Conservation of Nature and Natural Resources, Morges, Switzerland, 50 pp.

[B31] Wang Q Han X Xue XF Hong XY (2014) Three new species of eriophyoid mites (Acari, Eriophyoidea) associated with Lauraceae in China. ZooKeys 406: 81–100. doi: 10.3897/zookeys.406.6897 24843283 10.3897/zookeys.406.6897 PMC4023248

[B32] Watt G (1898) The pests and blights of tea plant. Government Press, Calcutta, India, 400–408.

[B33] Watt G Mann HH (1903) The pests and blights of the tea plant. 2nd ed Office Supplement Government Printing, Calcutta, India, 368–371.

[B34] Wei SG Feng YB Huang NX (1999) Three new species of Phyllocoptinae (Acari: Eriophyidae) from China. Systematic and Applied Acarology 4: 143–147. doi: 10.11158/saa.4.1.21

[B35] Zhang ZQ Fan QH Pesic V Smit H Bochkov AV Khaustov AA Baker A Wohltmann A Wen T Amrine JW Jr Beron P Lin J Gabrys G Husband R (2011) Order Trombidiformes Reuter, 1909. In: Zhang ZQ (Ed.) Animal biodiversity: An outline of higher-level classification and survey of taxonomic richness. Zootaxa 3148: 129–138. 10.11646/zootaxa.3703.1.1 26146682

